# Percutaneous pulsed radiofrequency treatment of the splanchnic nerves for chronic flank pain secondary to non-obstructive nephrolithiasis

**DOI:** 10.1016/j.inpm.2025.100541

**Published:** 2025-01-16

**Authors:** Edward Kim, Ratan K. Banik

**Affiliations:** Department of Anesthesiology, School of Medicine, University of Minnesota, Minneapolis, MN, USA

**Keywords:** Splanchnic nerve, Pulsed radiofrequency, Flank pain, Nephrolithiasis

## Abstract

Chronic benign flank pain of unknown etiology presents a significant challenge for pain physicians, especially when interventional treatment options are limited. We report the case of a 26-year-old male with a history of chronic left flank pain, recurrent non-obstructing nephrolithiasis, and a complex urologic background, who was referred to pain management after failing to find relief through previous urologic interventions. Despite the absence of new obstructing stones, the patient's pain persisted. Initial splanchnic nerve blocks using 0.25 % bupivacaine and dexamethasone provided temporary relief, leading to the decision to proceed with pulsed radiofrequency (RF) treatment of the left splanchnic nerves. Under fluoroscopic guidance, the pulsed RF procedure resulted in significant pain reduction, which lasted for four to six months. Over the course of four years, the procedure was repeated six times, providing sustained relief and allowing the patient to resume normal activities, including school and work. This case highlights the potential effectiveness of pulsed RF as a viable option for managing refractory chronic flank pain when other treatments have failed.

## 1 Introduction

One of the most challenging and enigmatic issues in chronic flank pain management is symptomatic non-obstructing nephrolithiasis, for which no standard of care currently exists. The absence of a clear physiological basis for these symptoms often leads to extensive diagnostic evaluations, tertiary consultations, and referrals to pain specialists or psychiatrists. Pain specialists frequently attribute such symptoms to musculoskeletal etiologies, recommending physical therapy—a treatment with limited efficacy in this patient population. Consequently, many patients are prescribed analgesics or, regrettably, dismissed as exhibiting drug-seeking behaviors, given the prevailing assumption that non-obstructing renal stones should not cause pain. This clinical presentation is so prevalent that it has been colloquially termed “small stone syndrome” [1]**.** While some urologists propose flexible ureteroscopy [2] and stone removal as therapeutic options, these interventions often fail to achieve pain relief, underscoring the necessity for alternative approaches.

Splanchnic nerve block may represent a promising therapeutic avenue for these cases [3]. Pain signals from the kidney and ureter are transmitted via afferent Aδ and C sympathetic fibers through the renal and mesenteric plexuses to the splanchnic nerves. Based on this anatomical understanding, techniques such as laparoscopic renal denervation have historically been employed to address renal or ureteral pain [4,5]**.** In the recent years, percutaneous radiofrequency (RF) ablation of splanchnic nerves has been introduced as a minimally invasive alternative for managing a variety of abdominal pain syndromes [3,6,7]**.** Unlike surgical interventions, percutaneous RF ablation is less invasive, can be performed under local anesthesia with mild sedation, and is associated with minimal adverse effects. More recently, for non-cancer pain, pulsed RF treatment has been successfully developed. This is a minimally invasive, non-neurodestructive pain treatment and unlike conventional RF ablation, pulsed RF provides pain relief without causing tissue destruction. Moeschler and colleagues reported effective pain relief using pulsed RF of the splanchnic nerves in a patient with loin pain hematuria syndrome [8], highlighting the potential of this technique for addressing renal-related pain syndromes.

To the best of our knowledge, the application of splanchnic nerve pulsed RF as a therapeutic intervention for symptomatic non-obstructive nephrolithiasis has not been documented in the existing medical literature. This underscores the novelty of our approach and highlights the potential for further exploration of this minimally invasive technique as a viable option for managing this challenging and poorly understood clinical condition.

## 2 Case report

Informed consent was obtained from the patient for the publication of this case report and accompanying images. A 26-year-old male with a history of depression, anxiety, and attention-deficit/hyperactivity disorder was referred to the pain clinic by his urologist for management of chronic left flank pain. The patient has a significant urologic history characterized by recurrent kidney stones and chronic left flank pain. At age five, he underwent a left pyeloplasty for ureteropelvic junction obstruction. At 12 years old, he experienced his first kidney stone, leading to repeated hospitalizations and numerous urologic interventions. A left ureteral stent was placed at age 13, followed by treatments such as extracorporeal shock wave lithotripsy, ureteroscopy, and stent replacements. Brushite stones composed 90 % of his calculi. Despite intermittent but severe pain, frequent flare-ups significantly disrupted his daily life, forcing him to leave college. When urologic procedures and opioid therapies failed to control his symptoms, he was referred to pain management for further evaluation and care.

At presentation, the patient described his pain as a constant, numb ache localized to the left flank, with a visual analog scale (VAS) pain score of 6–8 out of 10. No specific exacerbating or alleviating factors were identified. Physical examination revealed tenderness to light palpation in the left flank region. His medication regimen included acetaminophen 650 mg and ibuprofen 600 mg as needed, and for more severe pain, ketorolac 10 mg every 8 hours or oxycodone 5 mg every 4 hours as needed. Previous therapies included lidocaine ointment and patches, gabapentin, and cyclobenzaprine. The patient was also on sertraline and receiving regular psychotherapy, as his pain often exacerbated his anxiety.

**Fig. 1:**
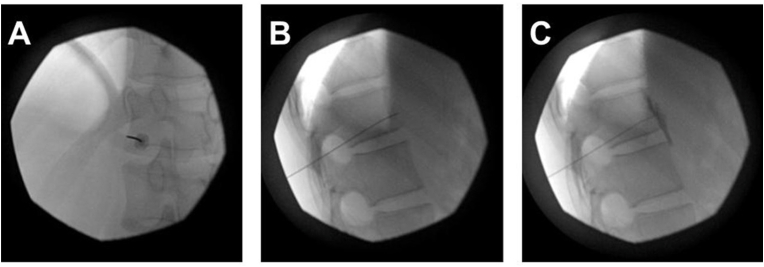
Multiplanar views of the splanchnic block procedure at the T11 level. These images show an anterior-posterior view (a), lateral view (b), and lateral view after contrast (c) of the thoracic spine using fluoroscopy. The anatomical target site was identified using fluoroscopy by aligning the superior endplate of T11 and then tilting the C-arm intensifier to position the tip of the T11 transverse process at the anterolateral border of the T11 vertebral body. The C-arm was then tilted upwards to move the 12th rib out of the way. The target was identified over the left lower part of the T11 vertebral body (A). A local anesthetic was administered by creating a skin wheal and inserting a 25-gauge, 1.25-inch needle to reach the hub. A 22-gauge, 100 mm spinal needle was then carefully guided just below the 11th rib. The needle was carefully advanced with continuous contact with the vertebral body, and multiple anterior-posterior and lateral fluoroscopy images (B) were taken to ensure the needle remained close to the vertebral body. The needle tip was advanced until it was just in front of the front edge of the T11 body (B). After confirming no aspiration, 2 cc of Isovue contrast was injected under live fluoroscopy, which showed excellent spread in the area in front of the vertebral body (C).

**Fig. 2:**
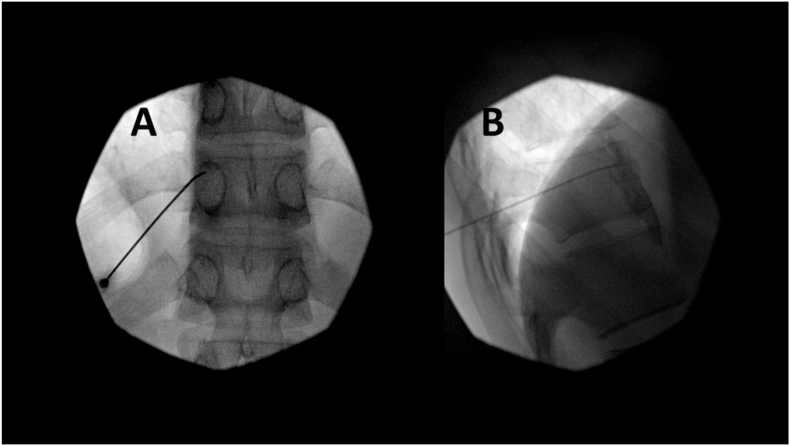
Multiplanar views of the splanchnic pulsed radiofrequency treatment procedure at the T11 level are presented. These images include an anterior-posterior view (a) and a lateral view (b), as detailed in Fig. 1.

A diagnostic and therapeutic left splanchnic nerve block (Fig. 1) was performed, involving the injection of 14 mL of 0.25 % bupivacaine with 1 mL of dexamethasone (4 mg). This intervention provided 75–100 % pain relief for 5 weeks. A second block further reduced his pain to an average VAS score of 1/10, with relief lasting 8 weeks. Given the encouraging results, the patient was scheduled for unilateral pulsed RF treatment of the left splanchnic nerves (Fig. 2) at the T11 vertebral body level to achieve longer-lasting pain control.

The anatomical target for pulsed RF was identified using fluoroscopic guidance. The superior endplate of the T11 vertebral body was squared off, and the C-arm was rotated obliquely to align the transverse process with the anterolateral border of the vertebral body. A 20-gauge, 150-mm curved-tip needle with a 10-mm active tip was introduced caudal to the costovertebral joint and advanced until positioned just ventral to the anterior border of the vertebral body. Injection of 2 mL of Isovue contrast confirmed excellent prevertebral spread without intravascular runoff. Sensory testing at 50 Hz induced severe flank pain at 2V, consistent with the patient's chronic pain pattern. Pulsed RF was performed at 2 Hz with a pulse width of 20 ms for 90 seconds, maintaining a maximum needle temperature below 42 °C. This procedure was repeated two times during the session, followed by the injection of 14 mL of 0.25 % bupivacaine and 1 mL of dexamethasone, confirming appropriate contrast dissipation on fluoroscopic imaging.

A single treatment resulted in substantial pain relief lasting nearly six months, during which the patient experienced significant functional improvement, attending school and work without absences. He had repeated this procedure six times over the four years.

## 3 Discussion

Single unilateral pulsed RF treatment of the left splanchnic nerves provided significant and sustained pain relief for four to six months in our patient with chronic flank pain secondary to non-obstructive nephrolithiasis. Over the course of four years, the procedure was repeated six times, providing sustained relief and allowing the patient to resume normal activities, including school and work. This outcome aligns with the findings of Moeschler et al., who reported a similar duration of benefit in a patient with loin pain hematuria syndrome treated bilaterally with pulsed RF [8]. During the effective treatment period, the patient experienced a substantial reduction in emergency department visits and chronic opioid medication usage. Upon recurrence of flank pain, he proactively sought a repeat pulsed RF procedure, which was repeated six times so far.

Flank pain is defined as discomfort in the area between the rib cage and ilium, often extending posteriorly from the midaxillary line [9]. Obstructing urolithiasis leading to renal colic is a well-documented etiology of flank pain, with resolution typically achieved following stone removal. However, patients with flank pain but without evidence of obstruction—attributed instead to small, non-obstructing nephrolithiasis—pose a diagnostic and therapeutic challenge [1]. Previous studies have demonstrated that small renal stones, despite lacking obstruction, could act as pain generators, with ureteroscopic intervention offering partial or complete symptom resolution in some cases [2]. Splanchnic nerve block and RF ablation have been previously described for the management of chronic abdominal pain conditions. Raj and colleagues first demonstrated the efficacy of RF ablation in treating chronic abdominal pain from various etiologies, including chronic pancreatitis and postsurgical abdominal syndromes, reporting substantial pain reduction in 107 patients [10,11]. Subsequent studies confirmed the utility of RF ablation in patients with abdominal pain from chronic pancreatitis [3,7] and pancreatic cancer [6].

We propose pulsed RF of the splanchnic nerves [12] as a minimally invasive alternative for managing non-cancer pain associated with non-obstructive nephrolithiasis. To our knowledge, there are no published reports describing the use of pulsed RF specifically for the management of chronic flank pain attributed to non-obstructive nephrolithiasis. Moeschler et al., however, reported effective pain relief using pulsed RF of the splanchnic nerves in a patient with loin pain hematuria syndrome [8].

The splanchnic nerves transmit nociceptive signals from abdominal viscera, including the kidneys and ureters [8]. Sympathetic innervation involves preganglionic fibers from T5-T12, which converge into the greater, lesser, and least splanchnic nerves [8]. These nerves, located in the anterolateral paravertebral space, offer a reliable target for pain interventions, providing an alternative to celiac plexus blocks. Notably, the least thoracic splanchnic nerve bypasses the celiac plexus [12], potentially allowing for more comprehensive coverage of renal sensory input when targeting the splanchnic nerves. Unlike celiac plexus neurolysis, which carries risks of complications due to the use of neurolytic agents such as alcohol or phenol, splanchnic pulsed RF offers a safer, minimally invasive option with fewer side effects.

Despite its benefits, splanchnic RF, either pulsed or ablation, raises concerns about masking clinically significant urolithiasis, particularly in patients with a history of obstructive stones. Undetected obstruction could lead to severe complications if early symptoms are blunted by pain management strategies. Additionally, splanchnic nerve RF is technically challenging, as the proximity of the target to the pleura which increases the risk of pneumothorax [3]. Proper patient selection is therefore critical to avoid concealing underlying serious pathology.

## 4 Conclusion

The management of chronic flank pain without a clear obstructive etiology, such as in cases of small, non-obstructing nephrolithiasis, remains a significant clinical challenge. This case highlights the potential of pulsed RF of the splanchnic nerves to provide meaningful, sustained pain relief while reducing reliance on opioids and improving quality of life. These findings suggest that splanchnic nerve pulsed RF may offer a promising therapeutic avenue for patients with non-obstructive nephrolithiasis as the suspected source of their pain. Further studies are warranted to validate these results and establish this approach as a viable treatment option for this difficult-to-manage condition.

## Disclosures

The authors declare no conflicts of interest.

## Declaration of competing interest

The authors declare no financial interests/personal relationships.
